# Review of the New American Cancer Society Guidelines for Breast Cancer Screening for Women at Average Risk

**Published:** 2016-07-01

**Authors:** Sylvia S. Estrada

**Affiliations:** Cedars-Sinai Medical Center, Samuel Oschin Comprehensive Cancer Institute— Brandman Breast Center, Los Angeles, California

Breast cancer is the most common cancer in women worldwide (Torre et al., 2015). In the United States, an estimated 231,840 women were diagnosed with breast cancer in 2015 (Siegel, Miller, & Jemal, 2015). After lung cancer, breast cancer continues to rank second as a cause of cancer death in women in the United States as well as remains the leading cause of premature mortality for women. Even though death from breast cancer has declined steadily since 1990, largely due to improvements in early detection and treatment (Berry et al., 2005), an estimated 40,290 women in the United States died of breast cancer in 2015 (Siegel et al., 2015).

In 2003, the American Cancer Society (ACS) issued its prior recommendations for breast cancer screening. The recommendations included annual mammography screening for all women starting at age 40 years and continuing as long as a woman remains in good health. Clinical breast examinations (CBEs) should be periodically performed for women in their 20s and 30s and annually for women 40 years and older (Smith et al., 2003).

Since the 2003 ACS recommendations, new evidence has accumulated from long-term follow-up of randomized controlled trials and observational studies of organized, population-based screening programs. There is also greater emphasis on estimating the harms associated with screening; assessing the balance of benefits and harms; and supporting the interplay among values, preferences, informed decision-making, and recommendations (Oeffinger et al., 2015). The ACS has incorporated standards recommended by the Institute of Medicine into its guidelines development protocol to ensure a more trustworthy, transparent, and consistent process for developing and communicating guidelines (Brawley et al., 2011).

## DEFINING AVERAGE RISK

The new guideline addresses recommendations for women at average risk. Average risk is defined as those women without a personal history of breast cancer, a confirmed or suspected genetic mutation known to be associated with increased risk (e.g., *BRCA*), or a history of radiotherapy to the chest at a young age (Oeffinger et al., 2015). These investigators acknowledged there are also women outside these higher-risk categories who are at higher-than-average risk of breast cancer. Mammography screening alone may have reduced effectiveness in these women. These higher-than-average risk women are those with significant family histories but without a high probability of carrying identified risk mutations, those with a prior diagnosis of benign proliferative breast, and those with significant mammographic breast density.

Intermediate-risk women may benefit from additional breast imaging other than screening mammography. At this time, the investigators have noted there are no reliable estimates of the number of women who have one or more of these risk factors; nor are there widely accepted risk-based screening recommendations that differ for women in this intermediate-risk group compared with average-risk women.

## REVIEW OF THE LITERATURE: EVIDENCE SYNTHESIS

The ACS organized an interdisciplinary guidelines development group (GDG) consisting of clinicians, biostatisticians, epidemiologists, an economist, and patient representatives to develop the new ACS guidelines for women at average risk (Oeffinger et al., 2015). The ACS GDG selected the Duke University Evidence Synthesis Group to conduct the independent systematic evidence review of the breast cancer screening literature. Formulation of recommendations was based on the quality of the evidence and judgment (incorporating values and preferences) about the balance of benefits and harms.

The evidence-based breast cancer screening guideline for women at average risk focused on three key questions out of the five key questions (see Table). Key points from the evidence synthesis follow:

- Screening mammography in women aged 40 to 69 years is associated with reduction in breast cancer deaths across a range of study designs; inferential evidence supports breast cancer screening for women aged ≥ 70 years who are in good health.
- Estimates of the cumulative lifetime risk of false-positive examination results are greater if screening begins at a younger age, due to the greater number of mammograms as well as to the higher recall rate for younger women. The quality of evidence for overdiagnosis is not sufficient to estimate a lifetime risk with confidence.
- Analysis of the effects of the screening interval indicates more favorable tumor characteristics when premenopausal women are screened annually vs. biennially.
- Evidence does not support routine clinical breast examination as a screening method for women at average risk.

**Table:**
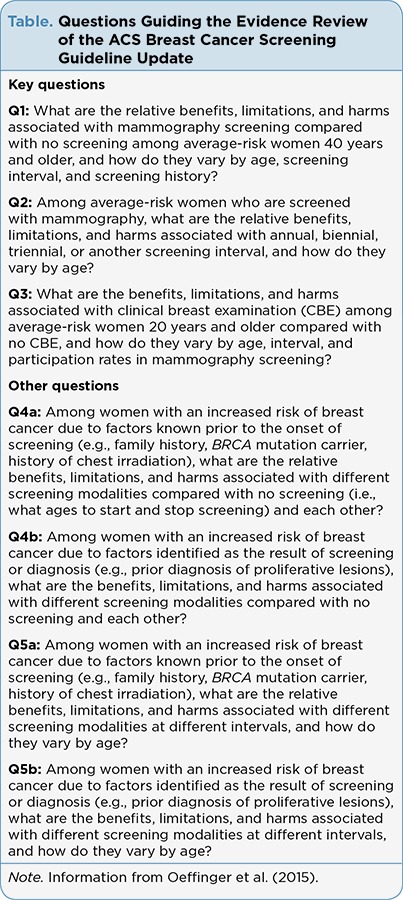
Questions Guiding the Evidence Review of the ACS Breast Cancer Screening Guideline Update

## UPDATED RECOMMENDATIONS

The updated recommendations are based on the GDG’s consensus judgment about when the benefits of mammography screening clearly or likely outweigh the harms in a population of women at average risk. These recommendations are designated as either strong or qualified. A strong recommendation indicates consensus that the benefits of adherence to an intervention outweigh the undesirable effects that may result from screening. A qualified recommendation indicates clear evidence of benefit of screening but less certainty about the balance of benefits and harms or patient values and preferences. These factors could lead to different decisions about screening (Oeffinger et al., 2015).

**Recommendation 1**

Women with an average risk of breast cancer should undergo regular screening mammography starting at age 45 years (Strong Recommendation).

1a. Women aged 45 to 54 years should be screened annually (Qualified Recommendation).

1b. Women 55 years and older should transition to biennial screening or have the opportunity to continue screening annually (Qualified Recommendation).

1c. Women should have the opportunity to begin annual screening between the ages of 40 and 44 years (Qualified Recommendation).

**Recommendation 2**

Women should continue screening mammography as long as their overall health is good and they have a life expectancy of 10 years or longer (Qualified Recommendation).

**Recommendation 3**

The American Cancer Society does not recommend clinical breast exam for breast cancer screening among average-risk women at any age (Qualified Recommendation).

**Limitations**

The investigators acknowledged there were inevitable gaps between the available evidence and the evidence needed for the development of guidelines that precisely quantify and weigh the benefits vs. the harms associated with breast cancer screening. These gaps need further research to help women make screening decisions. Better evidence about the extent of overdiagnosis is important, as is more information about the preferences and decision processes of diverse populations (Keating & Pace, 2015).

## CONCLUSION

The new ACS recommendations are made in the context of maximizing reduction in breast cancer mortality and reducing years of life lost while minimizing the associated harms among the population of women in the United States. The ACS affirms that screening mammography saves lives. Fewer women will die of breast cancer as a result of early detection from routine screening mammography. The ACS also recognizes that the balance of benefits and harms will be close in some instances and that the spectrum of women’s values and preferences will lead to varying decisions. The intention of this new guideline is to provide both guidance and flexibility for women about when to start and stop screening mammography and how frequently to be screened for breast cancer (Keating & Pace, 2015).

Advanced practitioners need to understand the content and message in the new ACS breast cancer screening guidelines. When counseling average-risk women older than 40 years, it is important to remember and emphasize there is no single right answer to the question "Should I have a mammogram?" Instead, women should be supported in estimating and understanding their risk of developing breast cancer and exploring their values and preferences so health-care providers can help them make informed decisions.
